# The cell death‐related genes machine learning model for precise therapy and clinical drug selection in hepatocellular carcinoma

**DOI:** 10.1111/jcmm.18168

**Published:** 2024-03-17

**Authors:** Mingyang Du, Yonggang Qu, Lingshan Qin, Jiahe Zheng, Wei Sun

**Affiliations:** ^1^ Department of Radiology Shengjing Hospital of China Medical University Shenyang Liaoning China; ^2^ Department of clinical medicine China medical university Second Hospital Shenyang Liaoning China; ^3^ Department of clinical medicine Fourth Affiliated Hospital of China Medical University Shenyang China

**Keywords:** hepatocellular carcinoma, immunotherapy, programmed cell death, tumour heterogeneity, tumour microenvironment

## Abstract

Hepatocellular carcinoma (HCC) is the prevailing subtype of hepatocellular malignancy. While previous investigations have evidenced a robust link with programmed cell death (PCD) and tumorigenesis, a comprehensive inquiry targeting the relationship between multiple PCDs and HCC remains scant. Our aim was to develop a predictive model for different PCD patterns in order to investigate their impact on survival rates, prognosis and drug response rates in HCC patients. We performed functional annotation and pathway analysis on identified PCD‐related genes (PCDRGs) using multiple bioinformatics tools. The prognostic value of these PCDRGs was verified through a dataset obtained from GEO. Consensus clustering analysis was utilized to elucidate the correlation between diverse PCD clusters and pertinent clinical characteristics. To comprehensively uncover the distinct PCD regulatory patterns, our analysis integrated gene expression profiling, immune cell infiltration and enrichment analysis. To predict survival differences in HCC patients, we established a PCD model. To enhance the clinical applicability for the model, we developed a highly accurate nomogram. To address the treatment of HCC, we identified several promising chemotherapeutic agents and novel targeted drugs. These drugs may be effective in treating HCC and could improve patient outcomes. To develop a cell death feature for HCC patients, we conducted an analysis of 12 different PCD mechanisms using eligible data obtained from public databases. Through this analysis, we were able to identify 1254 PCDRGs likely to contribute to cell death on HCC. Further analysis of 1254 PCDRGs identified 37 genes with prognostic value in HCC patients. These genes were then categorized into two PCD clusters A and B. The categorization was based on the expression patterns of the genes in the different clusters. Patients in PCD cluster B had better survival probabilities. This suggests that PCD mechanisms, as represented by the genes in cluster B, may have a protective effect against HCC progression. Furthermore, the expression of PCDRGs was significantly higher in PCD cluster A, indicating that this cluster may be more closely associated with PCD mechanisms. Furthermore, our observations indicate that patients exhibiting elevated tumour mutation burden (TMB) are at an augmented risk of mortality, in comparison to those displaying low TMB and low‐risk statuses, who are more likely to experience prolonged survival. In addition, we have investigated the potential distinctions in the susceptibility of diverse risk cohorts towards emerging targeted therapies, designed for the treatment of HCC. Moreover, our investigation has shown that AZD2014, SB505124, LJI308 and OSI‐207 show a greater efficacy in patients in the low‐risk category. Conversely, for the high‐risk group patients, PD173074, ZM447439 and CZC24832 exhibit a stronger response. Our findings suggest that the identification of risk groups and personalized treatment selection could lead to better clinical outcomes for patients with HCC. Furthermore, significant heterogeneity in clinical response to ICI therapy was observed among HCC patients with varying PCD expression patterns. This novel discovery underscores the prospective usefulness of these expression patterns as prognostic indicators for HCC patients and may aid in tailoring targeted treatment for those of distinct risk strata. Our investigation introduces a novel prognostic model for HCC that integrates diverse PCD expression patterns. This innovative model provides a novel approach for forecasting prognosis and assessing drug sensitivity in HCC patients, driving a more personalized and efficacious treatment paradigm, elevating clinical outcomes. Nonetheless, additional research endeavours are required to confirm the model's precision and assess its potential to inform clinical decision‐making for HCC patients.

## 1 INTRODUCTION

Hepatocellular carcinoma (HCC) is the commonest types of primary liver cancer. It arises from the main type of liver cells called hepatocytes and make up about 75% of liver cancer cases. Other types of primary liver cancer include cholangiocarcinoma, angiosarcoma and hepatoblastoma, but these are less common.^1^ According to epidemiological studies, primary liver cancer is the fourth most common malignant cancer in China, following lung cancer, gastric cancer and oesophageal cancer.^2^ Scientific evidence indicates that the onset of HCC, although its origins remain obscure, is closely associated with the use of alcohol, diabetes, non‐alcoholic steatohepatitis linked to obesity and hepatitis B virus infection as prominent risk factors.^3^ Early‐stage HCC often lacks specific symptoms, leading to delayed diagnosis and treatment. Additionally, non‐specific symptoms may be present, but they are not easily distinguishable from other medical conditions.^4^ As a result, timely recognition and diagnosis of HCC play a critical role in reducing the mortality rate associated with this disease.

Currently, the treatment options for HCC mainly include hepatectomy, radiofrequency ablation, radiotherapy, chemotherapy and targeted therapy.^5^ Nonetheless, the existing therapies are accompanied by substantial adverse effects, exorbitant costs and inter‐individual variations in efficacy. Furthermore, the pronounced incidence of post‐surgical recurrence and pharmacological resistance poses significant obstacles to HCC management and thrusts significant burdens on households and the community at large.^6^ Since tumours are extremely heterogeneous, patients with identical tumour stage undergoing the same course of treatment are expected to have distinct prognoses, with significant implications for patient outcomes. As a result, therapy regimens should be tailored based on the particular genetic and molecular characteristics that differ between tumours.^7^, ^8^ Given the heterogeneity of HCC tumours and the variation in treatment response among patients, there is a pressing need to explore new therapeutic modalities and develop personalized treatment regimens. This requires understanding the underlying genetic and molecular alterations driving tumour growth and progression, as well as identifying biomarkers that can predict treatment response and guide treatment decisions. By developing more targeted and individualized therapies, we can improve outcomes for HCC patients and ultimately reduce the burden of this disease.

Bioinformatics is a rapidly growing field that involves the use of computational tools and techniques to analyse biological data, including genomic, transcriptomic and proteomic data. In the field of medicine, bioinformatics is being increasingly used to identify potential disease gene targets, develop risk‐prognosis models and predict treatment responses. For example, bioinformatics is being used to analyse large genomic datasets to identify driver mutations or pathways in cancer, which can be targeted with specific therapies. Similarly, machine learning algorithms are being trained on patient data to develop models that can predict disease progression and guide treatment decisions. In this way, bioinformatics is playing an important role in advancing personalized medicine and improving patient outcomes.^9^, ^10^ A group of eight genes was identified as closely associated biomarkers of HCC, which were utilized to develop and validate relevant prognostic models. Moreover, an in‐depth analysis of the molecular biology underpinning this gene signature was undertaken to further elucidate its functional significance in HCC.^11^ A scientific investigation uncovered that genes associated with hypoxia may potentially serve as indicators for the diagnosis and prediction of prognosis of HCC. Additionally, predictive models were constructed.^12^ A research study has demonstrated the creation of a survival prediction model for HCC by m^6^A‐related genes. The model revealed that patients with varying risk scores exhibited dissimilar survival rates, TP53 mutation frequencies and responsiveness to targeted treatments.^13^ The aforementioned findings suggest that the application of bioinformatics analysis may provide innovative perspectives for the diagnosis and treatment of HCC.

Programmed cell death (PCD) is an evolutionarily conserved cellular suicide process that is crucial for regulating growth and development, maintaining tissue homeostasis and ensuring proper cellular function in multicellular organisms.^14^ PCD plays a prominent role in regulating cancer progression.^14^ Ferritin heavy chain (FTH), a ferroptosis‐related gene, plays an essential role in the modulation of iron homeostasis and is highly expressed in HCC cancer tissue samples. Studies suggest that increased expression of FTH may speed up the progression of HCC carcinogenesis.^15^, ^16^ A scientific investigation uncovered that the long non‐coding RNAs (lncRNAs) associated with cuproptosis were expressed at elevated levels in high‐risk HCC patients. The researchers further conducted experiments to confirm the aforementioned lncRNAs, potentially providing novel insights into the treatment of HCC.^17^ A recent report suggested that genes related to necroptosis could serve as potential independent prognostic factors, providing a novel target for individualized treatment of HCC.^18^ Despite the mounting evidence linking PCD to HCC, studies investigating multiple types of PCD are scarce. Therefore, we conducted a comprehensive analysis by integrating various genes associated with different PCD modalities to further explore the diagnosis and prognosis for HCC. Our approach aimed to provide novel biomarkers for the individualized treatment of HCC, which could improve patient outcomes and reduce mortality rates. By combining different PCD pathways and analysing their interactions, we hope to obtain a more comprehensive insight into the pathogenesis of HCC and develop effective therapies to combat this deadly disease.

## 2 MATERIALS AND METHODS

### 2.1 Data preparation and pre‐processing

To conduct our analysis, we used the RNA‐seq and clinical data from the TCGA‐HCC (*n* = 371, https://portal.gdc.cancer.gov/) and GEO‐GSE76427 datasets (115 primary tumours and 52 adjacent non‐tumour tissues, https://www.ncbi.nlm.nih.gov/geo/).^19^ After filtering out samples with missing data on survival time or survival status, our final dataset included 485 HCC samples.^20^ The integration of the two datasets was performed, and the R package ‘sva’ was utilized to mitigate batch effects. A comprehensive set of 1254 PCD‐related genes (PCDRGs) was derived from a previous study, encompassing diverse pathways (Table S1).^21^ TCGA and GEO allow researchers unrestricted access to and study relevant data without the need for additional ethical approval. We guarantee that our processes for obtaining and analysing data comply with relevant regulations.

### 2.2 Identification of prognostic PCDRGs

The initial step was to filter out PCDRGs with differential expression on the TCGA‐HCC dataset by the following criteria: |log_2_FC|≥1.5 and FDR < 0.05.^22^ After filtering out differentially expressed PCDRGs, we performed univariate Cox analysis to identify prognostic PCDRGs based on *p* < 0.01. These prognostic PCDRGs were further examined for their copy number variation (CNV) landscape to determine possible alterations in their expression due to CNVs. Additionally, we explored the mutual expression relationships between these PCDRGs to identify possible interactions that may affect patient prognosis.^23^, ^24^

### 2.3 Consensus clustering analysis

Through the use of the *K*‐means algorithm, we performed consensus unsupervised clustering analysis by the ‘ConsensusClusterPlus’ package in *R*.^25^ We based the screening criteria for *K* values on small intra‐group differences, large inter‐group differences and large sample size in each group. This allowed us to determine the optimal number of clusters for stratifying HCC patients according to their PCDRG expression patterns and ultimately predicting their prognosis.^26^ To further understand the potential clinical implications of the PCD clusters, we performed survival analysis and chi‐squared tests to identify any significant survival differences between the different clusters. Additionally, we utilized principal component analysis (PCA), t‐distributed stochastic neighbour embedding (tSNE) and Uniform Manifold Approximation and Projection (UMAP) to investigate the distribution and separation of the PCD clusters. Finally, we investigated the relationship between the different PCD clusters and clinical characteristics such as age, gender, tumour stage and treatment response to determine any potential associations or predictive capabilities of the clusters. The overall goal was to gain a comprehensive understanding of the prognostic relevance and clinical implications of the PCDRG‐based clustering of HCC patients.

### 2.4 Gene expression profile, immune cell infiltration and enrichment analysis

Our initial investigation involved a thorough analysis of the prognostic PCDRGs expression profiles across distinct PCD clusters. This enabled us to identify various PCD regulatory patterns associated with these genes.^24^ Next, we assessed the extent of immune cell infiltration in various PCD clusters using single‐sample gene set enrichment analysis (ssGSEA). We employed the Wilcox test to compare the differences in immune cell infiltration between the various PCD clusters. This analysis provided us with insights into the distinct immune landscape of each cluster.^27^ Lastly, we utilized the gene set variation analysis (GSVA) to evaluate the activation of Kyoto Encyclopedia of Genes and Genomes (KEGG) pathways in different PCD clusters. This analysis helped us identify the crucial pathways that were active in each cluster, which could potentially offer new perspectives into the pathogenesis of PCD. To visualize the results, we generated a heat map that displayed the top 30 enrichment results. This analysis further characterized the complex molecular landscape of PCD and provided new insights into the dysregulated pathways that drive this disease.

### 2.5 Construction of PCD prognostic model

Initially, the samples were allocated into the training and testing sets in a randomized manner, with a 7:3 ratio. Subsequently, a least absolute shrinkage and selection operator (LASSO) analysis was employed to further scrutinize the potential prognostic PCDRGs.^28^ Multivariate Cox analysis was applied to develop a model for PCDRG.^29^ To analyse the survival differences between the various risk groups, we employed survival analysis and the chi‐squared test. Furthermore, time‐dependent receiver operating characteristic (ROC) curves were utilized to evaluate the test efficiency of the model.^30^ We consider that an area under the curve (AUC) greater than 0.65 is a standard with good test efficiency. To determine if the model was an independent predictor of the prognosis of patients with HCC, we conducted both univariate and multivariate Cox analyses. This was done to assess the effect of the model's variables on the patient's survival outcomes while accounting for potentially confounding factors.

### 2.6 Development of a nomogram integrating model and clinical features

To investigate the distribution of risk scores within different PCD clusters, we analysed the data and plotted the outcomes. We then utilized a nomogram calibration plot to visualize the predictive values between survival events and the dummy observations. Within the nomogram that combined risk groups and clinical features, each variable was assigned a score, and these scores were totalled to produce a final overall risk score.

### 2.7 Immune landscape and immunotherapy

To quantify immune cell infiltration in the studied samples, we employed several algorithms including EPIC, MCP‐COUNTER, TIMER, XCELL, QUANTISEQ, CIBERSORT and CIBERSORT‐ABS. These algorithms are widely utilized in immune cell deconvolution analysis and are known to provide reliable estimates of immune cell types present in a given tissue or sample. Using these algorithms, we were able to assess the relative abundance of different immune cell types, which helped us better understand the tumour microenvironment (TME) and its potential impact on disease progression and treatment outcomes.^31^, ^32^, ^33^, ^34^, ^35^, ^36^, ^37^ Using ssGSEA, we evaluated the immune function in multiple risk groups and employed the Wilcoxon test to perform between‐group comparisons. Furthermore, we scrutinized the expression patterns of immune checkpoint genes (ICGs) across the diverse risk groups. Lastly, we utilized TMB scores as a predictive tool to forecast immunotherapy outcomes in HCC patients.^38^

### 2.8 Screening of chemotherapeutic agents and novel targeted drugs

By evaluating the activity of specific KEGG pathways across different risk groups, we aimed to identify potential drug targets for each group.^39^ After performing statistical analysis using the Wilcoxon test, we identified several significantly dysregulated KEGG pathways specific to each risk group, suggesting possible drug targets for each group. These findings provide insight into potential therapeutic options that may be tailored to specific risk groups in HCC patients.^40^

### 2.9 The assessment of the biological process

The *z*‐score technique was introduced with the aim of integrating feature gene expressions and emulating the activity of specific pathways.^41^, ^42^ The *z*‐score technique has been used to analyse a variety of gene sets, including angiogenesis, cell cycle, epithelial to mesenchymal transition (EMT) and PCD biomarkers.^43^, ^44^ The *z*‐score for each gene set represents the deviation of the observed gene expression levels from the mean expression level for that gene set. By calculating the *z*‐scores for multiple gene sets, researchers can compare the relative activity of different biological pathways and processes in a particular sample or condition.

### 2.10 Exploration of scRNA

To gain deeper understanding into the TME of HCC at the single‐cell level, we employed the TISCH2 database as a resource to analyse signature expression levels in individual cell populations of independent HCC patient cohorts through scRNA‐seq (GSE140228).^1^

### 2.11 Statistical analysis

All statistical analyses were conducted using R software (version 4.1.3). Various R packages including ‘gmodels’, ‘tidyverse’, ‘ggplot2’, ‘ggsci’ and ‘ComplexHeatmap’ were employed to generate the results. Statistical significance was determined by a *p* < 0.05 (*p < 0.05; **p < 0.01; ***p < 0.001; ****p < 0.0001).

## 3 RESULTS

### 3.1 Identification of PCDRGs

**FIGURE 1:**
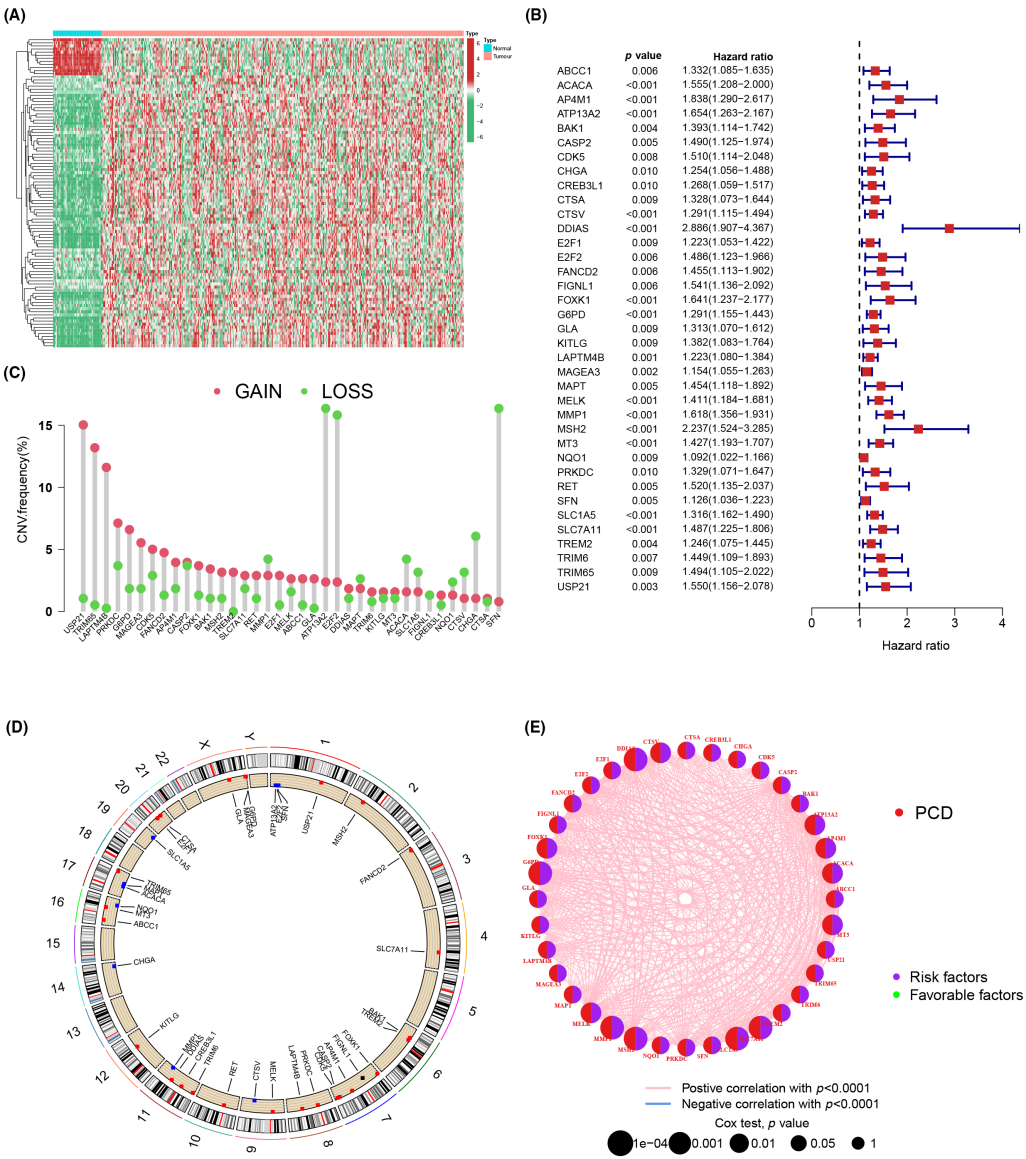
(A) Heatmap depicting differential expression of PCDRGs in normal and HCC samples. (B) Univariate Cox analysis for 37 PCDRGs in HCC patients, presented as a forest plot. (C) CNV frequency distribution of 37 PCDRGs in HCC. (D) Location of 37 PCDRGs on chromosomes. (E) Interaction analysis among 37 PCDRGs in HCC.

Figure 1A depicts the significant differential expression of PCDRGs in HCC and normal samples, as revealed via a heatmap. Prognostic PCDRGs were identified for HCC patients from TCGA via univariate Cox analysis, leading to the identification of 37 PCDRGs (Figure 1B). Subsequently, the CNV status of the 37 PCDRGs was analysed, revealing frequent copy number amplification in ABCC1, AP4M1, BAK1, CASP2, CDK5, CREB3L1, CTSA, DDIAS, E2F1, FANCD2, FOXK1, G6PD, GLA, KITLG, LAPTM4B, MAGEA3, MELK, MSH2, MT3, PRKDC, RET, SLC7A11, TREM2, TRIM6, TRIM65 and USP21. Alternatively, MMP1, ATP13A2, E2F2, MAPT, ACACA, SLC1A5, FIGNL1, NQ01, CTSV, CHGA and SFN exhibited a higher frequency of deletions than amplifications (Figure 1C). To further elucidate the mutations affecting PCDRGs, CNV locations on chromosomes were mapped (Figure 1D).

### 3.2 Classification of PCD‐related clusters in HCC

**FIGURE 2:**
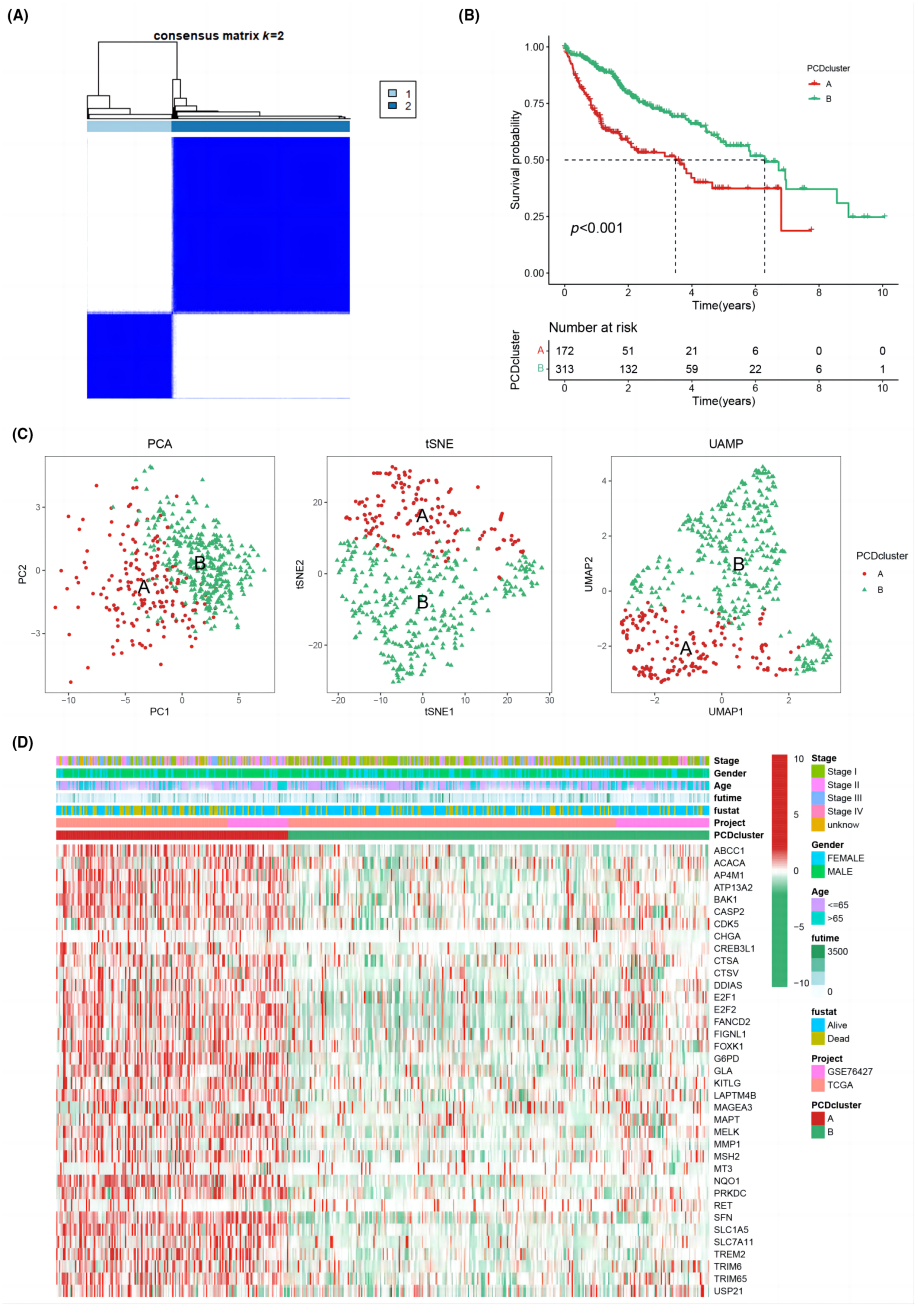
(A) Consensus matrix plot at optimal *k* = 2 for PCD clusters. (B) Kaplan–Meier survival analysis of PCD clusters. (C) PCA, tSNE, and UAMP plot for PCD clusters. (D) Differences in clinical characteristics and PCD expression levels for PCD clusters.

We conducted a screening of 485 HCC samples collected from the TCGA‐HCC and GEO‐GSE76427 datasets, with the aim of analysing the expression patterns of PCD genes. To facilitate our analysis, we constructed a PCD network that enabled us to identify interconnections among PCDRGs, as well as their regulators and prognostic values (Figure 1E). Using a consensus cluster method, we classified HCC samples by the expression profiles of 37 PCDRGs, and selected the optimal *K* value based on criteria including small differences within groups, large differences between groups and large sample sizes for each group. Our analysis suggests that *K* = 2 represents the most appropriate classification. Figure 2A and Figure S1 demonstrate that the entire cohort of HCC patients were divided into two PCDRG clusters (A and B) using a *k* value of 2. A Kaplan–Meier analysis was utilized to compare the two clusters, revealing that PCD cluster B had a significantly better survival probability (Figure 2B). Further analysis using PCA, tSNE and UMAP algorithms indicated significant differences between the two subtypes, strengthening the conclusion that the subtypes are reliable (Figure 2C). In addition, we observed significant differences between the two clusters of patients with respect to TNM stage, gender, age, survival time and survival status (Figure 2D).

### 3.3 Infiltration characteristics of TME in the distinct subtypes

**FIGURE 3:**
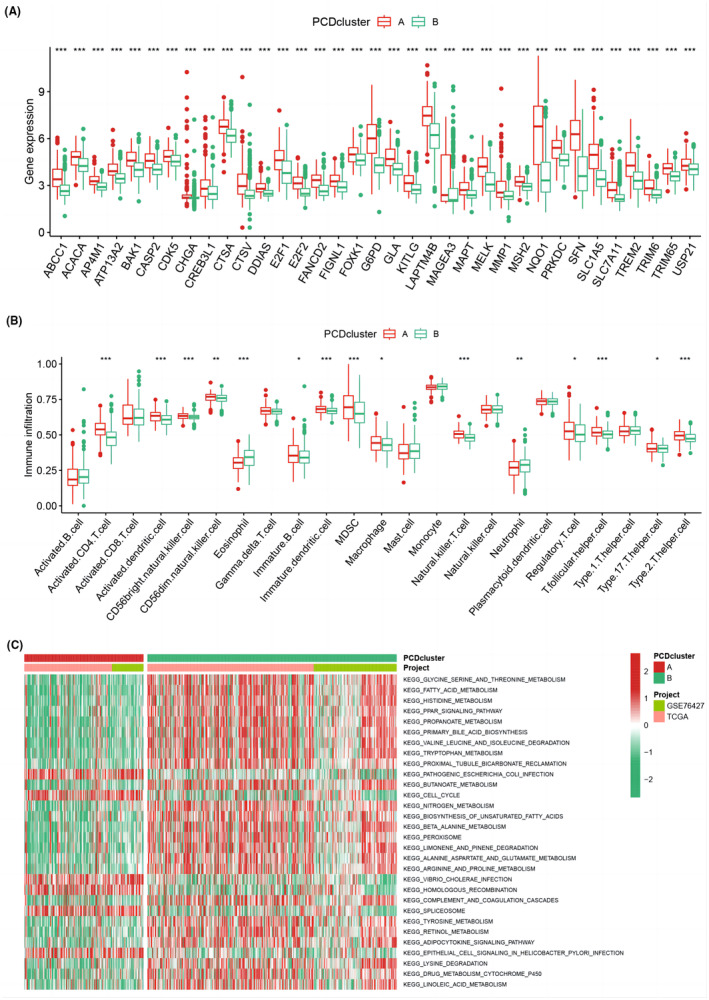
(A) Heatmap showing the expression levels of PCDRGs in PCD clusters. (B) Bar graph depicting the immune cell infiltration in PCD clusters. (C) GSVA enrichment analysis results for PCD clusters.

Using gene expression data, PCD cluster A had significantly higher expression of PCDRGs than PCD cluster B, indicating a closer relationship between PCD cluster A and PCDRGs (Figure 3A). Next, we investigated the immune cell infiltration in different PCD clusters using ssGSEA. The analysis revealed that neutrophils and eosinophils were more abundant in PCD cluster B (Figure 3B). Further analysis using GSVA showed that PCD cluster A was enriched in certain pathways, such as pathogenic *Escherichia coli* infection, cell cycle, *Vibrio cholerae* infection and homologous recombination, whereas PCD cluster B was enriched in pathways related to glycine, serine, and threonine metabolism, fatty acid metabolism, histidine metabolism and PPAR signalling pathway (Figure 3C).

### 3.4 Construction and verification of PCD signature

**FIGURE 4:**
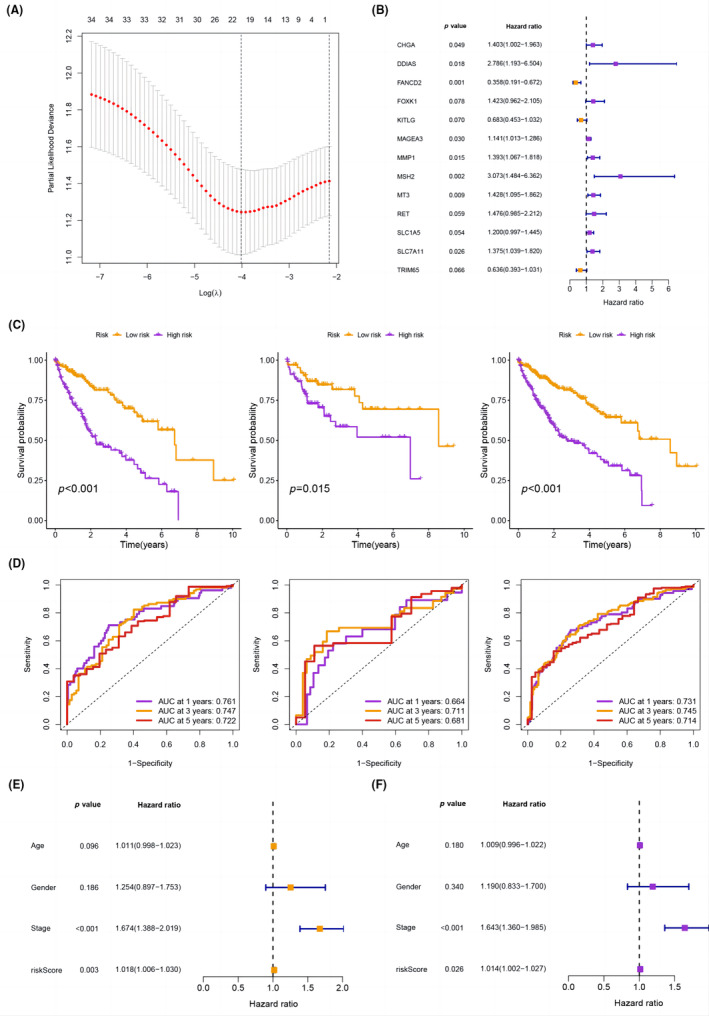
(A) Construction of a PCD‐based prognostic model using the LASSO algorithm for HCC patients. (B) A PCD prognostic model by multivariate Cox analysis. (C) Kaplan–Meier curves demonstrating the survival prognosis for the training, testing, and entire sets. (D) ROC curves for survival prognosis in the training cohort, testing cohort, and entire cohort. (E, F) Univariate and multivariate Cox analyses for prognosis of the PCDRGs signature.

To determine the optimal prognostic characteristics, we randomly allocated the samples to training and testing sets at a ratio of 7:3. Using LASSO and multivariate Cox analysis, we identified a 12‐gene signature that could predict HCC survival (Figure 4A,B). The PCD score was determined as a weighted sum of the expression values of the 12 genes: PCD score = (0.3383 × CHGA) + (1.0244 × DDIAS) + (−1.0274 × FANCD2) + (−0.3806 × KITLG) + (0.1321 × MAGEA3) + (0.3315 × MMP1) + (1.1226 × MSH2) + (0.3562 × MT3) + (0.3893 × RET) + (0.1825 × SLC1A5) + (0.3188 × SLC7A11) + (−0.4522 × TRIM65). The analysis suggests that FANCD2, KITLG and TRIM65 are low‐risk genes in HCC, while CHGA, DDIAS, FOXK1, MAGEA3, MMP1, MSH2, MT3, RET, SLC1A5 and SLC7A11 are high‐risk genes for HCC. The survival prognosis of the high‐risk group was significantly worse, as shown in Figure 4C. Moreover, we used time‐dependent ROC analysis to evaluate the predictive power of the prognostic model. The AUC values were 0.761/0.747/0.722 at 1/3/5 years in the training set, 0.664/0.711/0.681 at 1/3/5 years in the testing set, and 0.731/0.745/0.714 at 1/3/5 years in the whole set, indicating good predictive accuracy (Figure 4D). In addition, univariate Cox analysis revealed a remarkable association between the PCD score and the prognosis (hazard ratio (HR) = 1.018, 95% CI = 1.006–1.030, *p* = 0.003) (Figure 4E). Multivariate Cox analysis showed that the risk score of PCDRGs was an independent predictor of HCC prognosis (HR = 1.014, 95% CI = 1.002–1.027, *p* = 0.026) (Figure 4F).

### 3.5 Construction of a nomogram including model and clinical features for HCC

**FIGURE 5:**
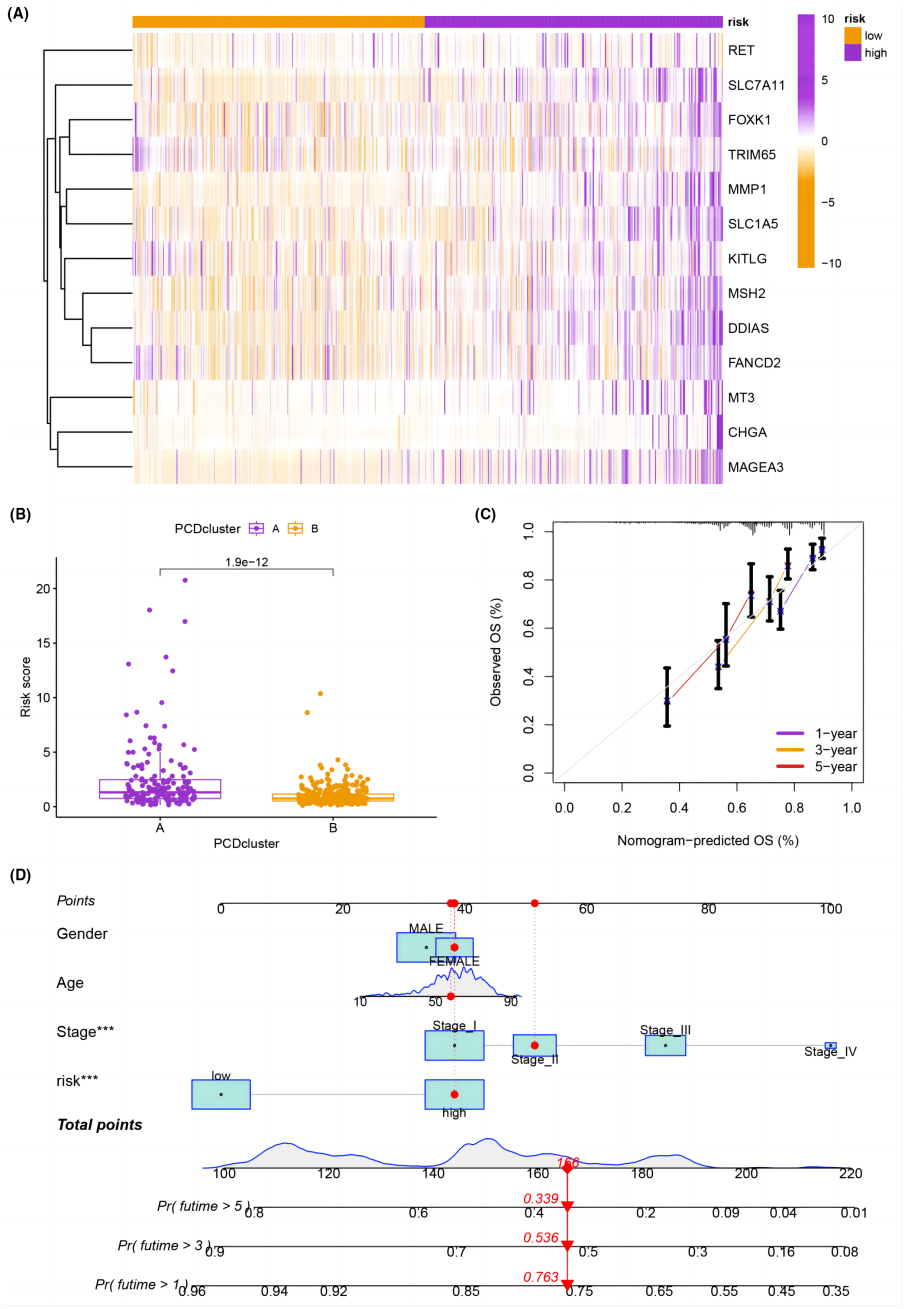
(A) Heatmap showing the expression levels of PCDRGs. (B) Bar graph displaying the difference in risk scores between the two PCD clusters. (C) Calibration curves for the prediction of survival probability at 1, 3 and 5 years. (D) Nomogram providing the predicted survival probability for HCC patients.

Figure 5A shows the expression levels of the 13 PCDRGs in the model. The PCD cluster A patients had higher risk scores (Figure 5B). We also constructed a nomogram combining the signature and clinical characteristics to predict the survival rates of HCC (Figure 5C,D). The nomogram was found to be reliable and sensitive in predicting survival.

### 3.6 Evaluation of immune landscape and immunotherapy

**FIGURE 6:**
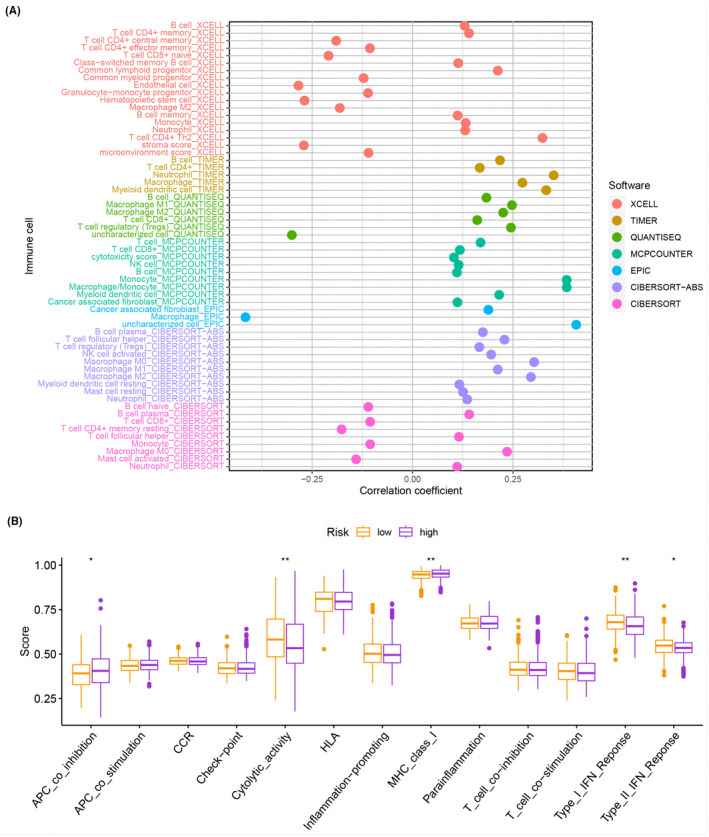
(A) Correlation between risk score and tumour‐infiltrating immune cells. (B) Differences in immune cells and immune function between high‐ and low‐risk groups.

**FIGURE 7:**
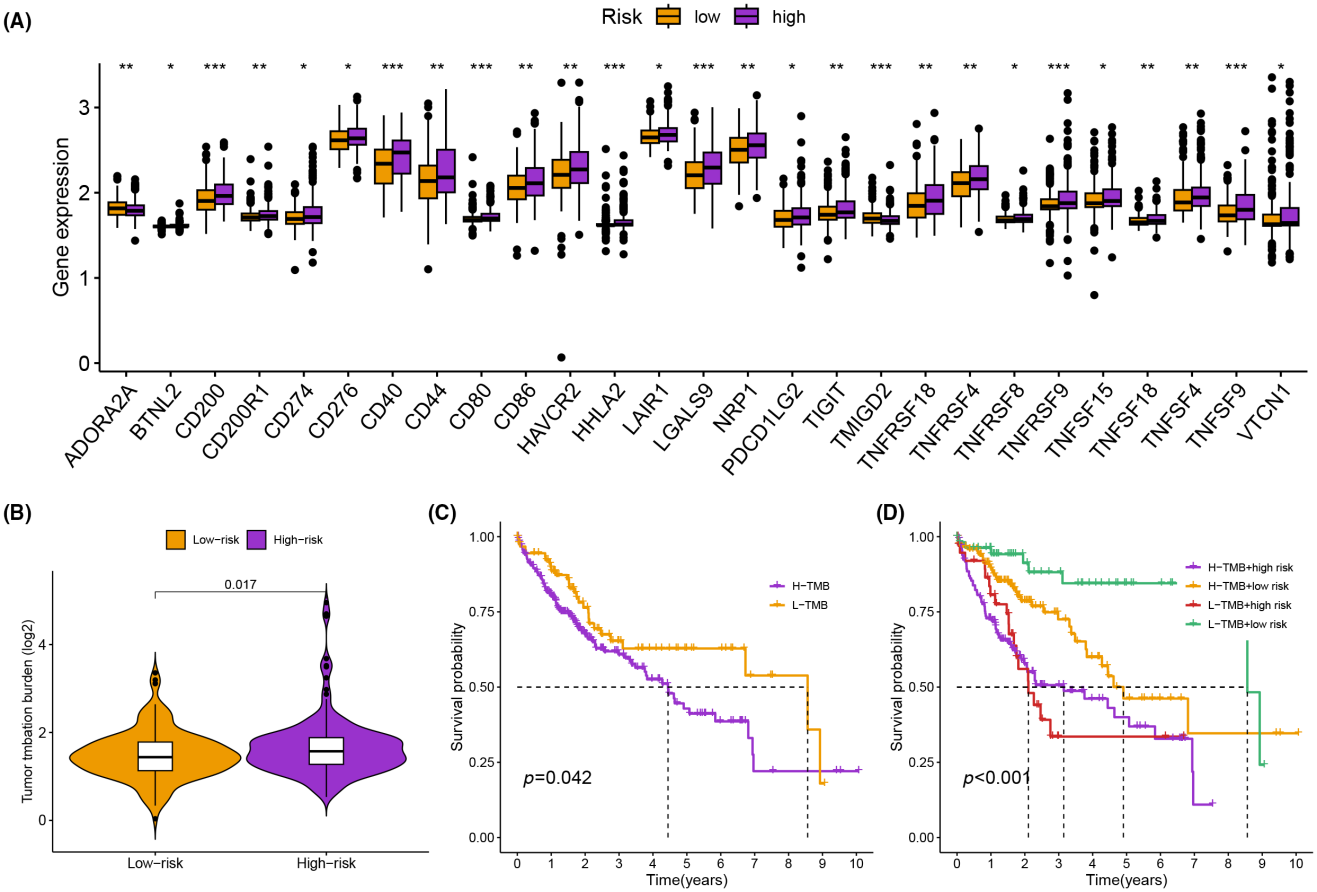
(A) Expression levels of ICGs in high‐ and low‐risk groups. (B) TMB scores in high‐ and low‐risk groups. (C) Kaplan–Meier analysis comparing subgroups based on TMB and risk score. (D) Kaplan–Meier analysis comparing four subgroups based on TMB and risk score.

Figure 6A and Table S2 present the associations between various immune cell types and risk groups. B cells, CD4^+^ memory T cells, CD4^+^ Th2 T cells, macrophages and neutrophils were positively correlated with high‐risk groups, while common myeloid progenitors, endothelial cells, haematopoietic stem cells and M2 macrophages were negatively correlated with high‐risk groups. Additionally, the ssGSEA results showed that cytolytic activity, Type I interferon (IFN) Response, and Type II IFN response were significantly upregulated in the low‐risk group, whereas APC co‐inhibition and MHC class I were notably upregulated in the high‐risk group (Figure 6B). In addition, we investigated the association between immune checkpoints and PCD‐related characteristics. We observed differential expression of 27 immune checkpoints (Figure 7A). The high‐risk group exhibited a significantly higher tumour mutation burden (TMB) (Figure 7B). The Kaplan–Meier curve revealed that patients with low TMB had a significantly higher survival probability (*p* = 0.042, Figure 7C). We combined the TMB group with the risk group to analyse the survival probability of different groups. The survival probability was highest for patients with low TMB and low‐risk (*p* < 0.001, Figure 7D).

### 3.7 Identified chemotherapeutic agents and novel targeted drugs

**FIGURE 8:**
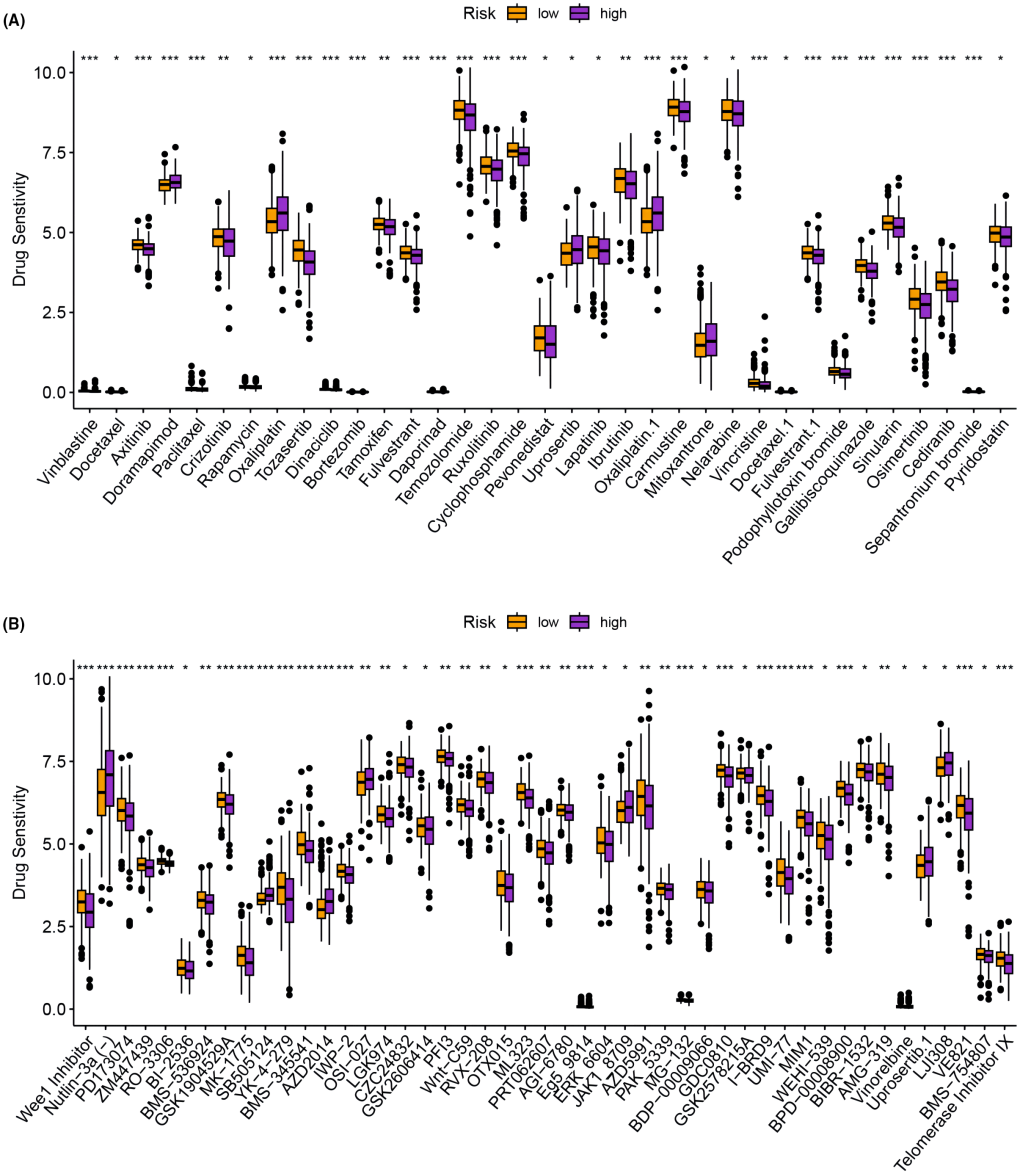
(A) Differential sensitivity of chemotherapy drugs in high‐ and low‐risk groups. (B) Differential sensitivity of novel targeted drugs in high‐ and low‐risk groups of HCC patients.

The low‐risk group showed greater sensitivity to Doramapimod, Oxaliplatin and Uprosertib among the screened chemotherapeutic agents, while the high‐risk group displayed greater sensitivity to Axitinib, Tozasertib, Sinularin and Osimertinib (Figure 8A). Additionally, we explored the sensitivity differences in novel targeted drugs between the various risk groups. We observed that the low‐risk group had better sensitivity to AZD2014, SB505124, LJI308 and OSI‐207, while the high‐risk group exhibited greater sensitivity to Wee 1 inhibitor, PD173074, ZM447439 and CZC24832 (Figure 8B). Therefore, our analyses suggest that the high‐risk group is more sensitive to both chemotherapeutic agents and novel targeted agents.

### 3.8 Evaluate the biological processes

**FIGURE 9:**
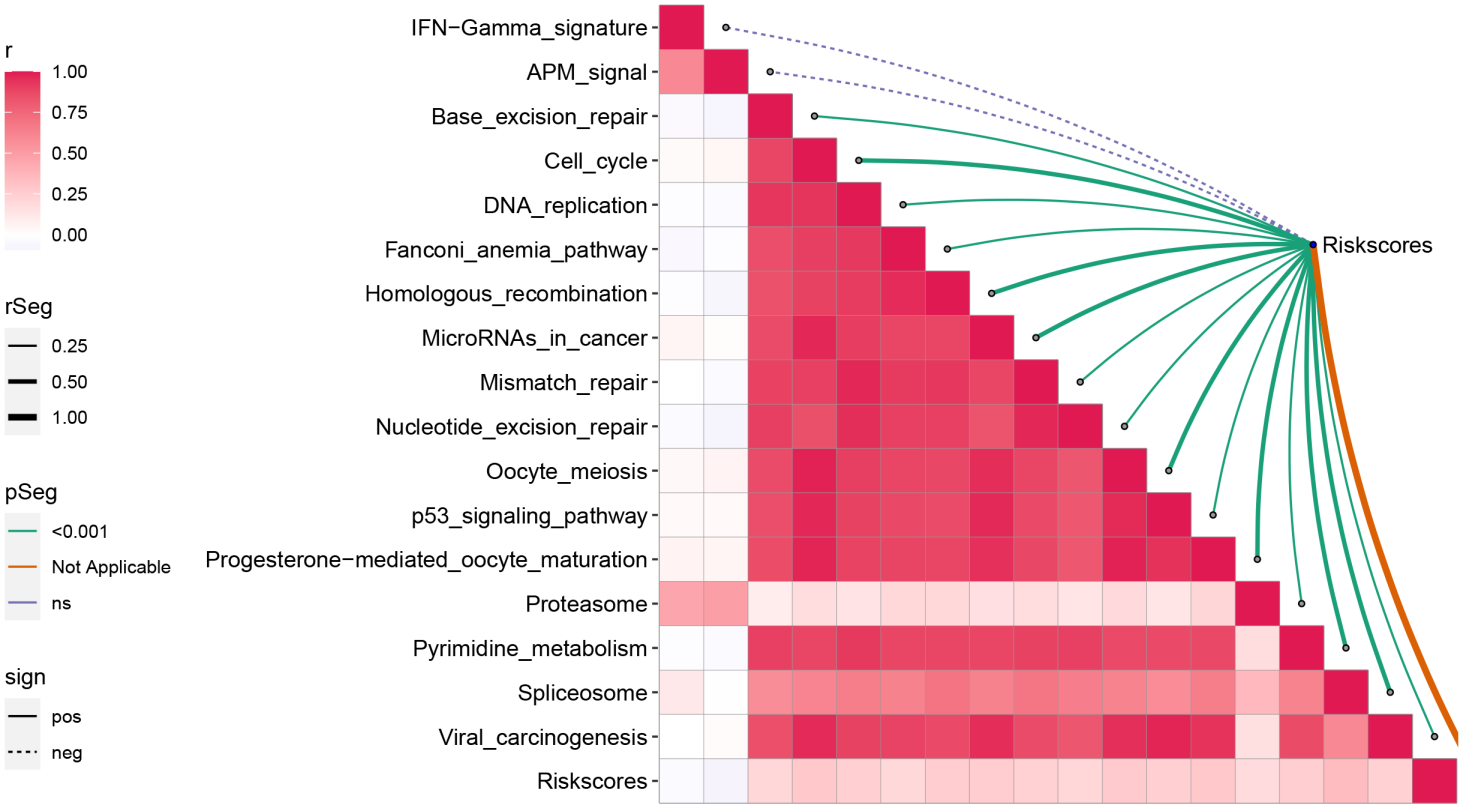
Correlation between risk score and the enrichment scores of immunotherapy‐predicted pathways.

Figure 9 demonstrates that the risk scores were inversely correlated with the KEGG signalling pathway including IFN‐Gamma signature and antigen processing and presentation (APM) signal, while positively correlated with the KEGG signalling pathway involving cell cycle, p53 signalling pathway, microRNAs in cancer and Oocyte meiosis.

### 3.9 Explore the relationship between PCDs and the malignant features of tumours

**FIGURE 10:**
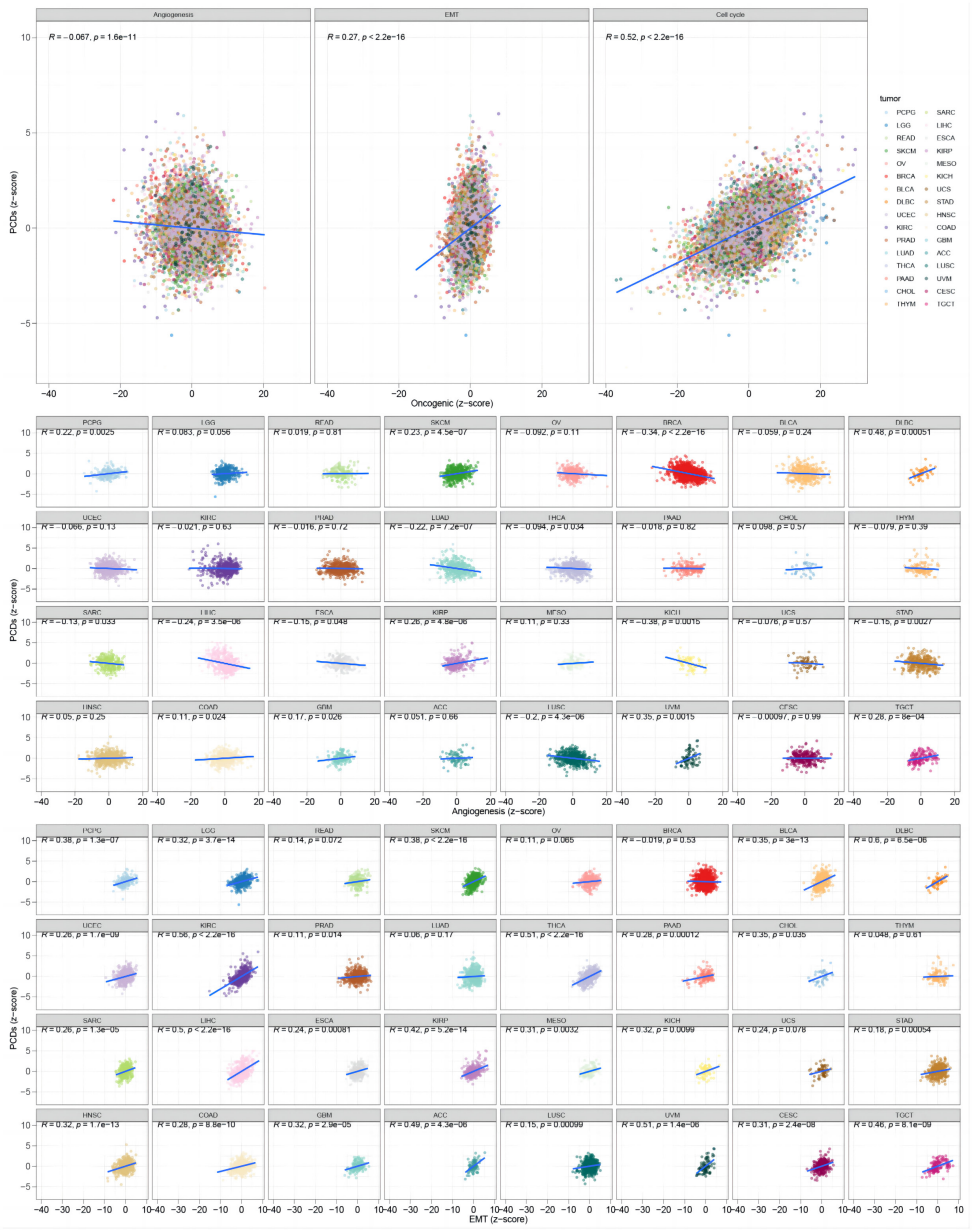
Association between PCD score and malignant features of the tumour.

Proliferation, invasion and metastasis, as well as promoting angiogenesis of tumour cells, have been linked to the development of HCC.^41^ PCD plays a critical role in the induction of cell death through complex signalling pathways and has been strongly linked to cancer progression and drug resistance.^45^ To examine the relationship between PCDs and the malignant features of cancer, we utilized the *z*‐score algorithm to determine the PCD, angiogenesis, EMT and cell cycle scores. In the TCGA pan‐cancer sets, there were significant correlations observed between the PCD *z*‐score and angiogenesis *z*‐score (*R* = −0.067, *p* < 0.0001), EMT *z*‐score (*R* = 0.27, *p* < 0.0001), as well as cell cycle *z*‐score (*R* = 0.52, *p* < 0.0001) (Figure 10).

### 3.10 Exploration of scRNA

**FIGURE 11:**
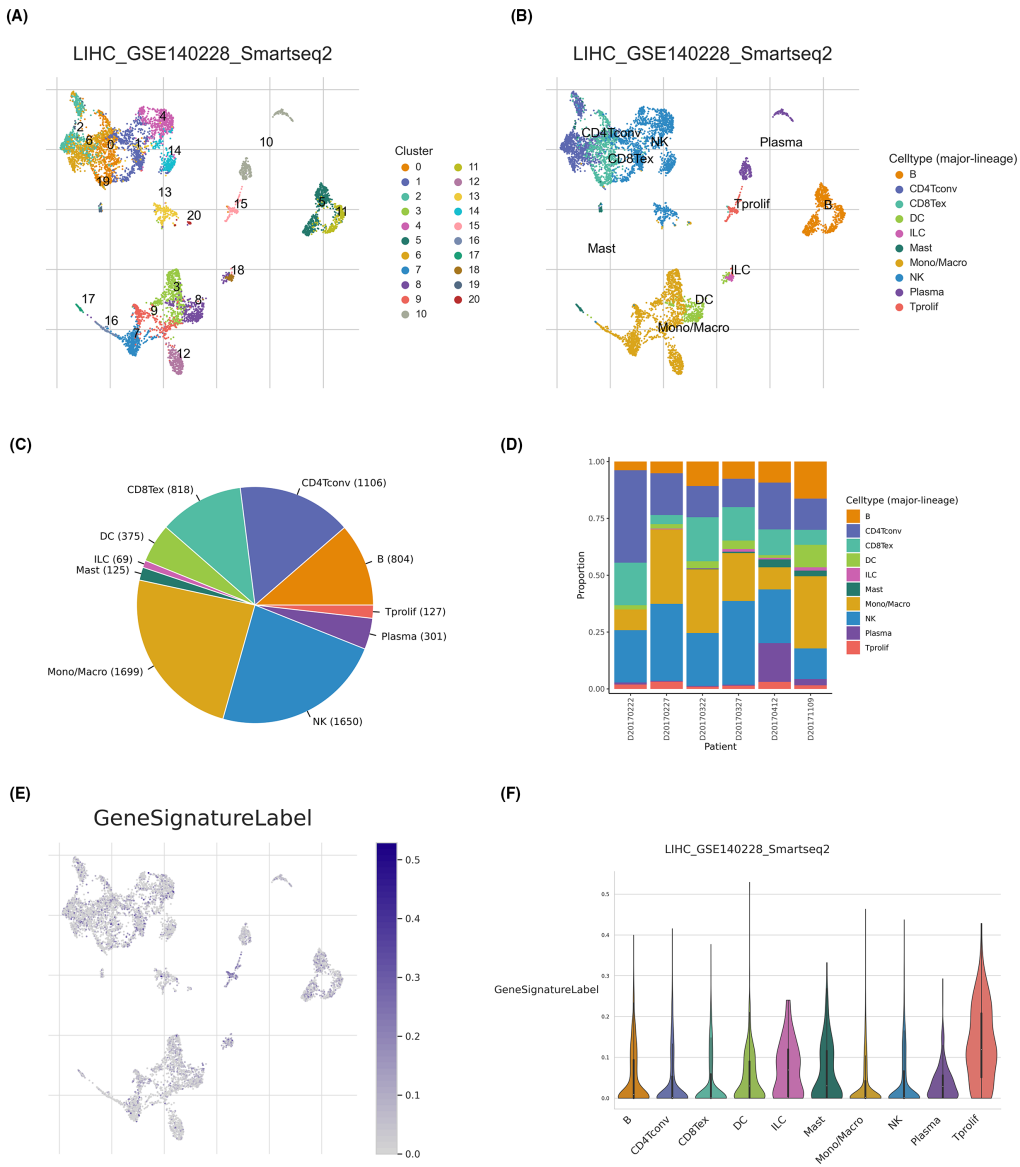
(A) The distribution of distinct clusters. (B) The distribution of diverse cell types. (C) The proportion of various cell types. (D) The proportion of different cell types across various samples. (E, F) Signature expression levels were significantly higher in T cell proliferation.

The results of scRNA‐seq are presented in Figure 11, where Figure 11A portrays the distribution of distinct clusters, Figure 11B illustrates the distribution of diverse cell types, Figure 11C exhibits the proportion of various cell types, and Figure 11D demonstrates the proportion of different cell types across various samples. The findings revealed a substantially higher expression of signature levels in T cell proliferation (Figure 11E,F).

## 4 DISCUSSION

HCC has become one of the major contributors to the global disease burden, with an increasing prevalence observed in recent years.^46^ Unhealthy dietary habits and liver virus infections are major drivers of the rising incidence of HCC in many countries. In 2020, China accounted for about 45.3% of all liver cancer cases and 47.1% of all liver cancer deaths in the world.^47^ Treatment plans for HCC depend on the patient's liver function status and the stage of the cancer, with options including radical treatment (surgery) and palliative treatment.^48^ However, the high‐risk of recurrence after treatment and individual differences in the response to treatment remain significant challenges in the management of HCC. Therefore, it is crucial to optimize existing treatment protocols and develop personalized management strategies for HCC.

PCD is integral to mammalian evolution; however, aberrant PCD can spur the advancement of carcinogenesis.^49^ Significant strides have been made in comprehending the mechanisms governing PCD, revealing that PCD is brought about by a cooperative interplay of various modalities and pathways. Apoptosis is a well‐ordered cellular process that eliminates damaged or expendable cells from an organism, characterized by chromatin condensation, cellular shrinkage and activation of cysteine aspartase, while avoiding a pro‐inflammatory state.^51^ In contrast to apoptosis, pyroptosis, an inflammatory member of PCD, is primarily regulated by the caspase family and featured by cellular swelling and rupture, followed by the release of high amounts of pro‐inflammatory cytokines that exacerbate the inflammatory response.^52^ Ferroptosis is an iron‐dependent PCD characterized with accumulation of excessive iron ions, disruption and crumpling of the outer mitochondrial membrane.^53^ Autophagy is a metabolic activity that involves the intracellular degradation of material in eukaryotic cells. In normal conditions, autophagy plays a role in sustaining intracellular homeostasis; however, disruption of the autophagic process may result in metabolic stress, degradation of cellular components and, potentially, PCD.^54^ Necroptosis is a form of PCD that is initiated when apoptosis is blocked and is mediated by the phosphorylation of MLKL by RIPK3. This process leads to cellular swelling, cytosol rupture and ultimately, the disintegration of the cytoplasm and nucleus.^54^ Cuproptosis is a PCD mechanism that involves the accumulation of intracellular copper.^55^ It occurs when copper combines with the lipidated constituents of TCA, resulting in abnormal protein aggregation, loss of iron–sulphur cluster proteins and proteotoxic stress that ultimately results in cell death.^56^, ^57^ Parthanatos is another form of PCD triggered by extensive DNA damage that activates PARP‐1 excessively.^58^ Entotic cell death occurs when one cell engulfs and kills another.^59^ Netotic cell death is characterized by the release of neutrophil extracellular traps.^60^ Lysosome‐dependent cell death, characterized by lysosomal rupture and typically mediated by the release of hydrolases or iron upon lysosomal membrane permeabilization, is another form of PCD.^50^ Alkaliptosis is triggered by intracellular alkalinization, while oxeiptosis is a type of PCD that is induced by oxidative stress and does not involve caspase activation.^50^, ^61^ Therefore, a detailed analysis of the 12 different forms of PCD can help to clarify the connection between PCD and HCC.

Overall, our study provides comprehensive analyses of the PCDRG expression profiles in HCC and identifies different PCD clusters that are associated with distinct clinical characteristics and immune cell infiltration patterns. Our PCD prognostic model, which includes 13 PCDRGs, may be a valuable tool for predicting the prognosis of HCC patients and improving clinical decision‐making. Future research is required to confirm and extend our results, and to investigate the potential mechanisms of PCD dysregulation in HCC tumorigenesis and progression. We quantified the relative abundance of immune checkpoint gene transcripts and used this information to prognosticate the efficacy of immunotherapeutic interventions in high‐risk patient populations. Furthermore, we conducted a systematic screening of candidate chemotherapeutic agents and novel targeted therapies for optimized treatment of HCC patients.

The TME comprises a complex milieu of cells, vasculature and signalling molecules that provide structural and biochemical support for tumour progression. The immune cells present in TME play a critical role in regulating inflammation and facilitating the tumour's ability to escape the immune system, in turn promoting tumour growth and invasion.^62^ Emerging evidence suggests that TME is involved in oncogenesis and cancer progression, and plays a crucial role in resistance to cancer therapy.^63^ Here, we report that PCD cluster B and high levels of eosinophils and neutrophils in this cluster are related to a better prognosis in HCC. Eosinophils, in particular, have multifunctional roles in the regulation of immunity and homeostasis and have been implicated in various stages of tumorigenesis and tumour progression.^64^ Studies in lung cancer have revealed that eosinophils are capable of releasing chemokines that attract anti‐tumour T cells from the immune system, thereby suppressing tumour growth.^65^ Additionally, eosinophils have also demonstrated anti‐tumour activity in HCC.^65^ Neutrophils,^66^ which are important in combating microbial infections, were previously believed to facilitate tumour progression by stimulating angiogenesis and metastasis.^67^ However, recent studies have shown that neutrophils might have potent anti‐tumour functions and help create an anti‐TME.^68^, ^69^ Targeting neutrophils for cancer treatment has also demonstrated significant clinical efficacy.^70^ Our study supports these previous findings, lending further credibility to our results.

Despite the promising results and reliability of our analysis, our study has some limitations that must be acknowledged. First, the data were obtained from public databases, which may be prone to bias and errors. Second, our sample size of HCC patients was relatively small, potentially leading to some inaccuracies in our analysis. Last, we did not conduct any experimental validation of the findings due to the difficulty in obtaining HCC tissues. Therefore, to provide stronger clinical evidence in HCC treatment, we plan to validate our analysis results through experiments in the future. Nonetheless, our research offers valuable insights into the prospective role of immune cells as biomarkers and therapeutic targets for HCC.

To summarize, our study demonstrates that the PCDRGs signature can serve as a reliable predictive tool for the prognosis of HCC. In addition, our analysis sheds light on the potential application of immunotherapy and drug sensitivity in the treatment of HCC. Collectively, our findings provide novel perspectives and strategies for improving the clinical management of HCC.

## AUTHOR CONTRIBUTIONS

**Mingyang Du:** Data curation (supporting); formal analysis (supporting); writing – original draft (lead); writing – review and editing (equal). **Yonggang Qu:** Data curation (equal); formal analysis (equal); writing – review and editing (equal). **Lingshan Qin:** Data curation (equal); formal analysis (equal); writing – review and editing (equal). **Jiahe Zheng:** Conceptualization (lead); writing – review and editing (lead). **Wei Sun:** Data curation (supporting); formal analysis (supporting); writing – original draft (lead); writing – review and editing (equal).

## CONFLICT OF INTEREST STATEMENT

The authors declare that they do not have conflict of interest.

## Supporting information


**Figure S1.** Consensus matrix for different *k* values (*k* = 3–9).

**Table S1.** List of PCDRGs.
**Table S2**. List of tumour‐infiltrating immune cells identified in this study.

## Data Availability

The data that support the findings of this study are available from the corresponding author upon reasonable request.
