# Supplementary figures and images for: The cell death‐related genes machine learning model for precise therapy and clinical drug selection in hepatocellular carcinoma
Source: J Cell Mol Med. 2024 Mar 17;28(7):e18168. doi: 10.1111/jcmm.18168 (PMC10945081; doi:10.1111/jcmm.18168)

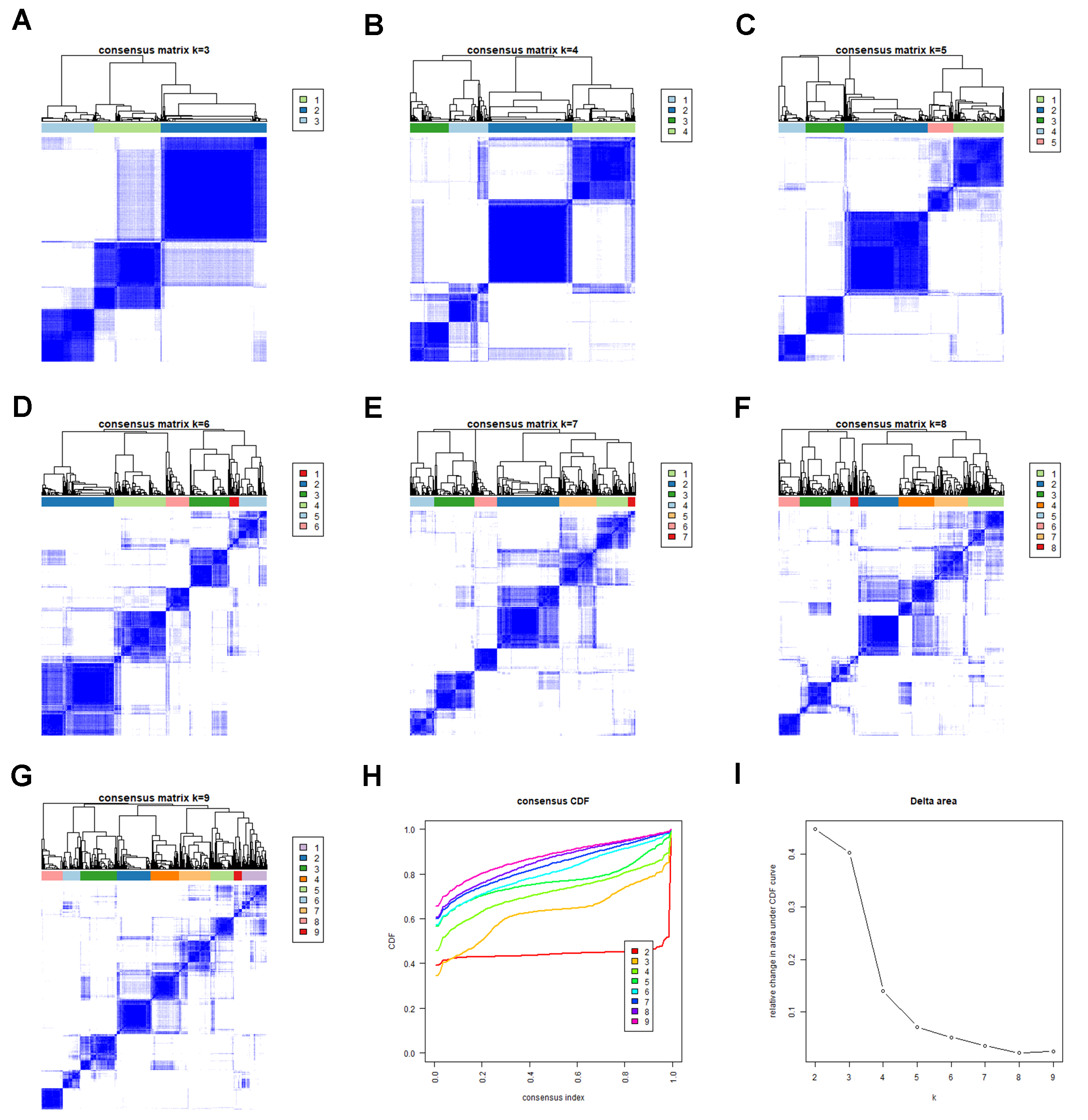

Supplement: Supplementary file 1 — Figure S1. Consensus matrix for different k values (k = 3–9). [file JCMM-28-e18168-s001.tif]
